# Expression pattern of miR-193a, miR122, miR155, miR-15a, and miR146a in peripheral blood mononuclear cells of children with obesity and their relation to some metabolic and inflammatory biomarkers

**DOI:** 10.1186/s12887-023-03867-9

**Published:** 2023-03-01

**Authors:** Maryam Behrooz, Samaneh Hajjarzadeh, Houman Kahroba, Alireza Ostadrahimi, Milad Bastami

**Affiliations:** 1 Department of Clinical Nutrition, School of Nutrition & Food Sciences, Tabriz University of Medical Sciences, Tabriz, Iran; 2 Pediatric Research Center, Tabriz University of Medical Sciences, Tabriz, Iran; 3 Student of Nutrition Sciences. Student Research Committee, Ahvaz Jundishapur University of Medical Sciences, Ahvaz, Iran; 4 Department of Toxicogenomics, GROW School of Oncology and Development Biology, Maastricht University, Maastricht, Netherlands; 5 Centre for Environmental Sciences, Hasselt University, Hasselt, Belgium; 6 Department of Clinical Nutrition, Nutrition Research Center, School of Nutrition & Food Sciences, Tabriz University of Medical Sciences, Tabriz, Iran; 7 Department of Medical Genetics, Tabriz University of Medical Sciences, Golgasht St, Attar Neyshabouri Av, Tabriz, Iran

**Keywords:** Obesity, MiRNAs, Children, Metabolic syndrome, Adolescents

## Abstract

**Background:**

The widespread presence of childhood obesity has increased considerably over three decades. The present study was designed to investigate expression patterns of miR-146a, miR-155, miR-15a, miR-193a, and miR-122 in peripheral blood mononuclear cells (PBMCs) in children who are obese along with their association with metabolic and inflammatory biomarkers.

**Methods:**

Ninety test subjects were admitted. The profile of blood pressure, resting energy expenditure (REE), anthropometric measures, body composition, dietary intakes, physical activity levels, insulin, and lipid profile, fasting blood glucose (FBG), high-sensitivity C-reactive protein (hs-CRP), and pubertal stage have been measured. Total RNA (including small RNAs) was extracted from PBMCs. The expression levels of miRNAs were measured by stem-loop RT-qPCR.

**Results:**

The miR-155a expression level was significantly lower in obese children, children with high hs-CRP, and children with high-fat mass. Obese girls had significantly higher PBMC levels of miR-122. MiR-155a had a significant negative association with fasting insulin, HOMA-IR, and hs-CRP. There were significant positive associations between miR-193a and miR-122 expression levels and fasting insulin, HOMA-IR, and TG. MiR-15a was positively correlated with fasting insulin and HOMA-IR. Children with metabolic syndrome, insulin resistance, and high-fat mass had higher PBMC levels of miR-122 and miR-193a. Higher miR-193a and miR-122 levels were also detected in PBMCs of children with fast REE, compared to those with slow REE, and the subjects with high hs-CRP, respectively.

**Conclusion:**

lower level of miR-155 expression in obese subjects and significant associations unfolds the need for more studies to detect the possible underlying mechanisms.

## Introduction

Obesity is a by-product of interactions among many environmental and genetic factors [1]. The common occurrence of childhood and adolescent obesity has seen a dramatic increase over the past three decades, turning it into a global health issue [2–4]. Obesity is strongly associated with insulin resistance, chronic adipose tissue inflammation, and hyperlipidemia. Undeniably, childhood and adolescent obesity increases the risk of chronic disorders such as cardiovascular diseases, dyslipidemia, metabolic syndrome, and diabetes mellitus in adulthood [5, 6]. While some obese children are metabolically healthy (MHO), others show abnormalities ranging from mild chronic inflammation to insulin resistance [7, 8]. It is estimated about 20 to 30% of obese children and adolescents are metabolically healthy [7, 9]. In most studies, metabolically healthy obesity has been suggested to be defined as obesity with no indication for associated metabolic disorders, such as dyslipidemia, metabolic syndrome. etc. [10]. Various hypotheses have been raised to explain the underlying mechanisms of this bipartite among obese children, either being metabolically healthy or suffering from obesity-related metabolic disorders, but more investigations are needed to achieve a consensus about the exact mechanism [11]. There is an urgent need for discovering biomarkers that help in the early detection and prognosis of high-risk individuals and the prevention of childhood obesity-related disorders [3]. MicroRNAs (i.e. miRNAs), recognized as promising biomarkers for various diseases [12]. are short non-coding molecules (about 22 nucleotides in length) that play crucial roles in regulating gene expression in the post-transcription step by binding to 3′-UTR-RNA regions, and inhibiting mRNA translation or degradation [2, 4, 13–15]. Being sustainable and measurable, circulating miRNAs in body fluids, especially blood, have been extensively used as a biomarker for the diagnosis of disorders [16–19]. In addition, several studies have suggested that circulating miRNA concentrations are associated with various metabolic diseases such as obesity, hyperlipidemia, and type 2 diabetes [20–22]. For example, a higher level of miR-122 is coincidental with the increased risk of obesity and insulin resistance in young adults [23, 24]. It correlates with an increased risk of metabolic syndrome, type 2 diabetes, and liver damage [25]. Previous studies have reported that miR-193 blood level was higher in obese and pre-diabetic adults [26]. In addition, miR-15 is reduced in type 2 diabetes, metabolic syndrome, and obesity, and is used as a biomarker to diagnose obese children at risk of type 2 diabetes henceforward [2]. MiRNAs are involved in adipogenesis, obesity, lipid and fatty acid metabolism, insulin resistance, adipocyte differentiation, appetite regulation, inflammation, oxidative stress, and cytokines expression [27, 28]. For example, miR-146 plays a crucial role in adipose tissue inflammation and is one of the most essential and effective mediators contributing to obesity and subsequent problems [29]. MiR-155 is involved in the metabolism of cholesterol and fatty acids in the liver and regulates many of the genes related to fat metabolism [30, 31]. It speculates that expression patterns of miRNAs in obese children could be used as a predictor of metabolic diseases in adulthood. miRNA expression profile of obese children, relative to others, has not been extensively investigated. The current study specified to compare the expression levels of miR-193a, miR-122, miR-155, miR-15a, and miR-146a in peripheral blood mononuclear cells (PBMCs) of 10 to 18 years-old obese children and adolescents with matched normal-weight subjects. Also, the association of these levels with metabolic conditions and inflammatory parameters is investigated.

## Methods

### Study subjects

First of all, the methodology and objectives of the study were explained to all of the participants and their parents; then they signed the informed consent form completely optional. 45 obese and 45 normal-weight 10 to 18 years old participants were involved in the present cross-sectional study. The criteria for matching the obese and normal-weight groups were the age and sex of the participants. Based on the 2–20 years old growth charts of CDC, children, and adolescents with a 5 ≤ BMI < 85 percentile and with a BMI ≥ 95th percentile were categorized as normal-weight and obese groups, respectively [32]. The exclusion criteria for the current study were unwillingness to enroll in the study for any reason, history of using medications including steroids, antiepileptic, and anti-psychotic medications, and history of chronic systemic diseases, including gastrointestinal, respiratory, cardiovascular, or neurologic disease, and being involved with obesity-related syndromes including Prader-Willi.

### Sample size

For sample size calculation, we used Mahdavi et al. study’s data [33]. Based on the reported correlation between mir-155 and BMI (r = -0.31). The final calculated sample size was 90 persons (refusal rate = 15%) α (two-tailed) = 0.05 β = 0.2 r = -0.31 *n* = 79.

### Physical and anthropometric variables

The puberty status of children and adolescents was evaluated using a provided form of the Tanner stages (consisting of the pictures of five stages). The research team clarified the form to the participants; then they were asked to choose the proper stage that portrayed their physique in the most proper way. Weight, height, hip circumference (HC), waist circumference (WC), BMI (Body Mass Index), and blood pressure (systolic and diastolic) were measured after fasting for 12 h overnight. Waist to hip ratio (WHR) was calculated. Weight (Kg) was divided by height (m) square for calculating BMI. Body composition was analyzed using Tanita MC-780 S MA (Amsterdam, the Netherlands). Physical activity levels of children and adolescents were assessed by MAQ (Modifiable Activity Questionnaire). A previous study on an adolescent population has reported moderate validity and high reliability for the Persian translation of the MAQ in this age range [34].

### Dietary assessment

To evaluate participants’ dietary intakes, trained interviewers used the 168 Quantitative Food Frequency Questionnaire. The validity and reliability of the questionnaire have been previously studied [35, 36]. Energy intake per day for every participant was calculated using Nutritionist IV software (version 3.5.2).

### Resting energy expenditure (REE) estimation

Indirect Calorimetry using Fitmate Pro., Rome, Italy was done to measure REE (VO2 and VCO2) between 06:00 to 08:00 AM. For more accuracy, doing exercise during the day before and eating any food from 21:00 the night before the visit was forbidden for all of the children and adolescents.

### Biochemical measurements

After overnight fasting of 12 hours, blood samples were taken. Levels of fasting insulin, FBG (Fasting Blood Glucose), lipid profile, and hs-CRP (high-sensitivity C - reactive protein) were measured for every participant. Enzymatic methods and colorimetry techniques were utilized for measuring serum HDL-C (High-Density Lipoprotein Cholesterol), TG (triglyceride), and FBG. The commercial kits for the calorimetry technique were provided by Pars-Azmoon Co., Tehran, Iran. ELISA method (Monobind, Lake Forest, CA, USA) was used to estimate insulin levels. Hyperinsulinemia was distinguished as following cut-off levels: higher than 30 μU/mL in the pubertal period, 20 μU/mL in the post-pubertal period, and 15 μU/mL in the pre-pubertal period [37, 38]. The equation below was used to calculate HOMA-IR [35]. (HOMA-IR= [fasting glucose (mg/dl) × fasting insulin (µIU/ml)]/405). Following cut-offs were considered for HOMA-IR to discern insulin resistance: ≥2.67 (specificity 65.5%, sensitivity 88.2%) and ≥ 2.22 (specificity 42.3%T sensitivity 100%) in boys and girls in the pre-pubertal stage respectively; ≥ 5.22 (specificity 93.3%, sensitivity 56%) and ≥ 3.82 (specificity 71.4%, sensitivity 77.1%) in boys and girls in the pubertal stage respectively [34]. Friedewald equation was employed for calculating LDL-C (Low-Density lipoprotein Cholesterol). Assessing hs-CRP levels in serum was done using the turbidimetric method. Based on gender and age, hs-CRP Reference values were spotted; Normal values in serum (mg/liter) including: (Boys: < 1.45 in 5–13 years old; < 2.13 in 14–18 years old) and (Girls: < 1.90 in 5–13 years old; < 3.33 in 14–18 years old). The subjects with MetS were diagnosed based on Cook et al. [39]. criteria, as follows: 1: HDL-C < 40 mg/dL. 2: TGs ≥ 110 mg/dL. 3: FBG ≥ 110 mg/dL . 4: WC ≥ 90th percentile for age and sex. 5: SBP and DBP ≥ 90th percentile for sex, age, and height .The subjects were categorized in the MetS group if they had at least three of the five mentioned criteria.

### miRNA extraction from PBMCs

A panel of 5 miRNAs that have previously been shown to correlate with obesity and markers of metabolic disease mainly in adults or experimental models was selected [2, 24, 26, 29, 31]. Using density gradient centrifugation (Ficoll-Paque PLUS, Amersham Pharmacia Biotech, Sweden), PBMCs were isolated from ~ 4 ml peripheral blood. Phosphate-buffered saline (PBS) was used for washing isolated PBMCs. Total RNA including small RNAs was extracted by Mini Kit of miRNeasy (Qiagen, Germany). The standard stem-loop miRNA RT-qPCR method [35] was employed to synthesize the first-strand cDNA of the studied miRNAs (hsa-miR-155-5p, hsa-miR-193a-3p, hsa-miR-15a-5p, hsa-miR-122-5p, hsa-miR-146a-5p). Reverse transcriptions were performed in 10 μl reactions and the following program: 30 min at 16 °C, 30 min at 42 °C, and 10 min at 75 °C Table 1. presents the sequence of primers that were exploited in reverse transcription and qPCR reactions.

**Table 1:** Sequence of primers that were utilized in reverse transcription and qPCR reactions

gene	Primer type	Primer sequence
**miR-155**	F	CGGCGCTTAATGCTAATCGTGATAG
	RT	GTCGTATCCAGTGCAGGGTCCGAGGTATTCGCACTGGATACGACACCCCT
	R	GTGCAGGGTCCGAGGT
**miR-146a**	F	GTGTGGGTGAGAACTGAATTCCATG
	RT	GTCGTATCCAGTGCAGGGTCCGAGGTATTCGCACTGGATACGACAACCCA
	R	GTGCAGGGTCCGAGGT
**miR-122**	F	GGCTGGAGTGTGACAATGGT
	RT	GTCGTATCCAGTGCAGGGTCCGAGGTATTCGCACTGGATACGACCAAACA
	R	GTGCAGGGTCCGAGGT
**miR-193a**	F	AGCGAACTGGCCTACAAAGTC
	RT	GTCGTATCCAGTGCAGGGTCCGAGGTATTCGCACTGGATACGACACTGGG
	R	GTGCAGGGTCCGAGGT
**miR-15a**	F	GGCGTAGCAGCACATAATGGT
	RT	GTCGTATCCAGTGCAGGGTCCGAGGTATTCGCACTGGATACGACCACAAA
	R	GTGCAGGGTCCGAGGT
**D24**	F	CGCTATCTGAGAGATGGTGATGACATT
	RT	GTCGTATCCAGTGCAGGGTCCGAGGTATTCGCACTGGATACGACTGCATCAG
	R	GTGCAGGGTCCGAGGT

### Quantitative real-time PCR

Light cycler 96 (Roche Diagnostics, Mannheim, Germany) was used to perform quantitative Real-Time PCR (qPCR) reactions using a 2 × qPCR master mix (RealQ Plus 2 × Master Mix Green without ROX, Ampliqon). Each 10 μl qPCR reaction contained the following components: 2 μl of forwarding primer (5 pmol/μl concentration), 0.5 μl of reverse primer, and, 5 μl master mix (RealQ Plus 2 × Master Mix Green without ROX, Ampliqon), 1 μl cDNA, 2 μl nuclease-free water and 1.5 μl dNTP. The qPCR reactions were performed in the following program: 15 min at 95 °C, followed by 40 cycles of 10 s at 95 °C and 30 s at 60 °C. At the end of qPCR cycles, the specificity of the reactions was evaluated by interpreting the melting curve step. To evaluate relative quantifications, the framework of Hellemans et al. Was used [40]. For a gene of interest (GOI) in each sample, ΔCq was equal to (mean Cq (GOI) in [all samples]) minus (Cq(GOI) in [that sample]). Then, relative quantities (RQs) were computed for each sample-GOI as RQ equals E(ΔCq), in which “E” was the mean efficiency of qPCR reactions for each gene. Normalized relative quantity (NRQ) for each sample-GOI was then calculated as NRQ (GOI) equals RQ (GOI) divided by the geometric mean of RQ values of the reference genes. NRQ values were converted to the Cq′ values by log2 transformation for the statistical analysis [41]. For normalizing miRNA quantities, the RQ value RNU24 was used. RNU24 is one of the stable small RNAs in PBMC which has previously been evaluated and used for normalizing miRNA quantities in PBMC in other studies [42, 43].

### Statistical analysis

SPSS Version 23.0 statistical software (SPSS Inc., Chicago, IL, USA) was used for statistical analysis. *P* < 0.05 was considered statistically significant. Quantitative variables are reported as mean (SD) or median (interquartile range [IQR]), as appropriate. Categorical variables are presented as numbers and percentages. Continuous variables that failed the normality test were logarithmically transformed before analysis. Non-parametric tests were used for the data that still did not have a normal distribution after this transformation. To compare differences between groups, independent samples T-test, Mann–Whitney U, or Kruskal–Wallis test were used. Spearman rank correlation coefficient (R) was used to test the association between selected miRNAs expression levels with some metabolic and inflammatory biomarkers.

## Results

Anthropometric, demographic, and laboratory characteristics of obese and normal-weight children were compared and the results were shown in Table2. There was no significant difference between the two groups in age, gender, and pubertal stage variables (*P* > 0.05); but the mean birth weight of obese children was significantly lower than normal-weight children (*P* = 0.03). As expected, the mean values of weight, BMI, waist circumference, hip circumference, and WHR of obese cases were higher than the control group (*p* < 0.001).

**Table 2:** Baseline demographic, anthropometric and laboratory characteristics of participants

Characteristics	Normal weight children (*N* = 45) Mean (SD)	Children with obesity (*N* = 45) Mean (SD)	^@^*P*-value
**Age (years)**	13.73(3.02)	13.92(2.98)	0.83
**Gender **^**$**^			
Boy	22(49)	22(49)	1.00
Girl	23(51)	23(51)	
**Pubertal stage **^**$**^			
Pre pubertal	7(15.5)	5(11.11)	0.84
pubertal	26(57.8)	24(53.33)	
Post pubertal	12(26.7)	16(35.56)	
**Birth weight (gr)**	3402.71(497.29)	3122.03(715.12)	**0.03**
**Weight (kg)**	48.57(16.81)	75.90(21.65)	** < 0.001**
**Height (cm)**	158.47(18.09)	160.00(12.92)	** < 0.001**
**BMI (kg/m**^**2**^**)**	18.57(3.02)	29.38(5.28)	** < 0.001**
**Hip circumference (cm)**	85.90(10.87)	101.56(21.95)	** < 0.001**
**Waist circumference (cm)**	71.40(12.68)	96.43(13.96)	** < 0.001**
WHR *	0.83(0.10)	0.91(0.11)	** < 0.001**
**Total Energy Intake**	1970.06(734.03)	2039.33(781.08)	0.42
**Physical Activity (MET-h/week)**	14.15(4.31)	15.03(5.25)	0.37
**WBC (10**^**9**^ /L) *	6.38(1.38)	6.97(1.11)	**0.01**
**RBC (1**^**6**^/mcL) *	5.01(0.44)	5.07(0.43)	0.58
**HB (gr/dL)**	13.29(1.48)	13.79(1.20)	0.13
**HTC (%)**	40.68(3.38)	41.54(3.07)	0.27
**MCV (fl)**	81.46(4.82)	82.02(3.87)	0.54
**MCHC(g/dL)**	26.93(2.20)	27.17(1.71)	0.62
**PLAT (10**^**3**^/μl) *	247.86(57.33)	272.3(45.79)	0.06

^@^Independent Sample Test or Mann–Whitney u Test for Quantitative variables and Chi-square test for categorical variables. *Median (IQR). ^$^N (%)

Obese children had significantly higher SBP, DBP, TC (*P* < 0.001), LDL-C, TG (*P* Z 0.001), Insulin, HOMA-IR, hs-CRP, and significantly Lower HDL-C (*P* < 0.001). The results related to these parameters are given in detail in our previous article [44].

Among laboratory indices, WBC count was higher in obese children in comparison to normal-weight children (*P* = 0.01). Considering the effect of WBC count on the miRNA’s expression levels, we divided obtained miRNAs expression levels by the average WBC count in each group (obese and normal children). So, selected miRNAs expression levels per unit of WBC count among children with and without obesity are estimated and presented in Table 3. Among the five studied miRNAs, the miR-155 expression level was significantly lower in obese children compared to the normal-weight group (*P*-value = 0.01). After disaggregating the data by gender, the miR-155 expression level difference was significant in girls (*P*-value = 0.03), but not in boys. Although the expression levels of miR-122 and miR-193a were higher in obese children than in normal-weight children, the differences were not statistically significant (*P*-values = 0.07). Considering the gender distribution, only the difference between obese and normal girls became significant for miR-122 (*P*-value = 0.04). The expressions of the other two miRNAs did not differ between the two groups Table 4. presents the association of the five studied miRNAs expression levels with serum levels of insulin, HOMA-IR, fasting blood glucose, hs-CRP, IL-1ß, IL-10, and lipid profile. There was a significant positive association between the expression level of miR-122 and fasting insulin, HOMA-IR, hs-CRP, and TG (*P* < 0.05). The expression level of miR-193a was associated with fasting insulin, HOMA-IR, and TG level (*P* < 0.05) significantly and directly. MiR-155 showed a significant negative association with fasting insulin, HOMA-IR, and TG level (*P* < 0.05). MiR15a expression had a significant association with fasting insulin and HOMA-IR, (P < 0.05) Table 5, 6, and 7.  present the comparison of miR-193a, mir122, and mir155 expression levels among children with and without some metabolic status (including obesity, metabolic syndrome indices, and different levels of REE, different body composition, hyper-insulinemia, insulin resistance, and high hs-CRP respectively. The median (IQR) expression level of miR-193a was significantly higher in children with metabolic syndrome, high TG, larger WC, insulin resistance, and high-fat mass (for a review of *P*-values between two groups, cf Table 5). There was also a significant difference in the expression level of miR-193a among children with slow REE and fast REE (*P* = 0.008). We couldn’t find any significant difference in miR-193a expression level of children with increased FBG, hyperinsulinemia, higher SBP/DBP, lower HDL, and higher hs-CRP with children who were in the normal spectrum for these biomarkers. Also, the expression level of miR-193a was not significantly different among children with different muscle mass.

**Table 3:** Selected miRNAs expression levels among children with and without obesity

**Gene** **NRQ**		**children with obesity**	**Normal weight children**	†***P*- value**
**miR-193a**	**Total**	1.01 (0.81)	0.64 (0.68)	0.07
	**Boy**	1.11(0.79)	0.49(0.31)	0.06
	**Girl**	1.00(0.98)	0.69(0.82)	0.40
**miR-122**	**Total**	0.21 (3.11)	0.06 (0.28)	0.07
	**Boy**	0.146(1.92)	0.06(0.16)	0.65
	**Girl**	0.86(1.53)	0.07(1.08)	**0.04**
**miR-155**	**Total**	**0.17 (0.43)**	**0.37 (0.61)**	**0.01**
	**Boy**	0.29(0.51)	0.47(0.66)	0.21
	**Girl**	0.06(0.15)	0.34(0.59)	**0.03**
**miR-15a**	**Total**	0.10 (0.29)	0.08 (0.29)	0.17
	**Boy**	0.12(0.55)	0.07(0.14)	0.14
	**Girl**	0.08(0.21)	0.09(0.52)	0.74
**miR-146a**	**Total**	0.09 (0.22)	0.10 (0.14)	0.66
	**Boy**	0.08(0.16)	0.08(0.12)	0.95
	**Girl**	0.12(0.51)	0.11(0.22)	0.70

^†^Mann–Whitney u Test,

*NRQ* Normalized relative quantity, *IQR* Inter Quartile Range

**Table 4:** The associations between expression levels of selected miRNAs and fasting insulin, HOMA-IR, fasting blood glucose, lipid profile and some inflammatory biomarkers in children

**Parameters**	miR-122 *r(*P*-value)	miR-193a *r(*P*-value)	miR-155 *r(*P*-value)	miR-15a *r(*P*-value)	miR-146a *r(*P*-value)
Insulin	**0.38(0.005)**	**0.40(0.003)**	**-0.37(0.001)**	**0.22(0.04)**	0.15(0.19)
HOMA-IR	**0.38(0.004)**	**0.40(0.004)**	**-0.36(0.001)**	**0.23(0.04)**	0.15(0.20)
FBS	0.09(0.44)	-0.14(0.22)	0.08(0.48)	0.05(0.65)	-0.08(0.49)
TG	**0.28(0.03)**	**0.38(0.04)**	0.15(0.18)	0.09(0.42)	0.07(0.56)
TC	0.05(0.67)	-0.02(0.80)	0.18(0.10)	-0.02(0.82)	-0.11(0.34)
HDL-c	-0.02(0.87)	-0.07(0.52)	-0.01(0.92)	-0.03(0.75)	-0.01(0.93)
LDL-c	-005(0.66)	-0.10(0.37)	0.16(0.16)	-0.02(0.80)	-0.10(0.43)
IL-10	-0.10(0.38)	-0.12(0.28)	-0.03(0.78)	0.02(0.84)	0.6(0.59)
IL-1ß	-0.06(0.61)	-0.14(0.22)	-0.11(0.31)	0.01(0.91)	-0.06(0.60)
hs-CRP	**0.28(0.03)**	0.22(0.20)	**-0.25(0.02)**	0.20(0.08)	0.04(0.69)

^*^Spearman rank correlation coefficient

F*BS* Fasting Blood Glucose, *TG* Triglyceride, *TC* Total Cholesterol, *HDL-C* High Density Lipoprotein Cholesterol, *LDL-C* Low Density Lipoprotein Cholesterol, *IL*-10 Interlukin-10, *IL*-1ß Interlukin-1ß, hs-*CRP* high-sensitivity *C* Reactive Protein

**Table 5:** The comparison of miR-193a expression level among children with and without some metabolic status

Groups	*N*	Median (IQR)	^@^*P*-value	Groups	*N*	Median (IQR)	^@^*P*-value
**Obesity**			0.07	^**^Muscle Mass Range			0.09
No	45	0.64(0.68)		Low	41	0.56 (0.71)	
Yes	45	1.01(0.81)		Normal	24	0.70 (0.59)	
**Metabolic syndrome**			**0.003**	High	25	1.13 (0.82)	
No	76	0.62(0.64)		^**^Fat Mass			**0.05**
Yes	14	1.31(0.42)		Normal	55	0.67(0.64)	
^**&**^**High TG**			**0.05**	high	35	1.11(0.85)	
No	50	0.64(0.65)		^**$$**^**REE**			**0.008**
Yes	40	1.06(0.76)		^*^Slow	10	^*^^1.11(0.49)	
^**&**^**Low HDL**			0.07	Normal	59	0.59(0.65)	
No	67	0.68 (0.64)		^*^Fast	21	^*^^1.61(0.49)	
Yes	23	1.21 (0.80)		^*^High hs-CRP			0.23
^**&**^**Large WC**			**0.01**	No	60	0.70 (0.88)	
No	67	0.64(0.64)		yes	29	0.85 (0.73)	
Yes	23	1.31(0.75)		^**%**^**Hyper-insulinemia**			0.38
^**&**^**High SBP**			0.32	No	80	0.70(0.84)	
No	80	0.70 (0.68)		Yes	10	0.80(0.87)	
Yes	10	1.08 (1.12)		^**#**^**Insuline Resistance**			**0.04**
^**&**^**High DBP**			0.37	**No**	76	0.70(0.72)	
No	39	0.70 (0.84)		**Yes**	14	1.13(1.10)	
Yes	51	1 (0.83)					

^@^Mann–Whitney u Test or Kruskal–Wallis test /^*P*-value = 0.05

^&^Normal Range according to metabolic syndrome definition in children

^**^Desired range reported in each person's body analyzer result sheet, was considered for classification of muscle mass (low-normal-high) and fat mass (low-normal-high)

^$$^ Classification for REE (slow-normal-fast) according to desirable ranges reported for each child in Indirect Calorimeter result sheet

^*^hs-CRP Reference Value according to sex and age. Normal value in serum (mg/liter): Boys: 5–13 year < 1.45 and 14–18 year < 2.13/Girls: 5–13 year < 1.90 and 14–18 year < 3.33

^%^Fasting insulin levels above 15 μU/mL in the pre-pubertal period, 30 μU/mL in the pubertal period and 20 μU/mL in the post-pubertal period are cut-off levels for hyper insulinemia

^#^ HOMA-IR cut-off values for insulin resistance were calculated to be 2.67 (sensitivity 88.2%, specificity 65.5%) in boys and 2.22 (sensitivity 100%, specificity 42.3%) in girls in the prepubertal period, and 5.22 (sensitivity 56%, specificity 93.3%) in boys and 3.82 (sensitivity 77.1%, specificity 71.4%) in girls in the pubertal period

**Table 6:** The comparison of miR-122 expression level among children with and without some metabolic status

Groups	*N*	Median (IQR)	^@^*P*-value	Groups	*N*	Median (IQR)	^@^*P*-value
**Obesity**			0.08	^**^Muscle Mass Range			0.13
No	45	0.06 (0.28)		Low	41	0.05(0.33)	
Yes	45	0.21(3.11)		Normal	24	0.16(2.00)	
**Metabolic syndrome**			**0.008**	High	25	0.21(3.95)	
No	76	0.06(0.33)		**Fat Mass			**0.04**
Yes	14	1.07(13.17)		Normal	55	0.06(0.25)	
^**&**^**High TG**			0.17	high	35	0.27(2.20)	
No	50	0.06 (0.88)		^**$$**^**REE**			0.09
Yes	40	0.22 (3.98)		^~^Slow	10	0.57(2.75)	
^**&**^**Low HDL**			0.27	^~^Normal	59	0.07(0.31)	
No	67	0.08 (0.88)		^~^Fast	21	0.09(18.32)	
Yes	23	0.22 (3.98)		*High hs-CRP			**0.04**
^**&**^**Large WC**			**0.03**	No	60	0.06(0.70)	
No	67	0.21 (1.02)		Yes	29	0.31(3.07)	
Yes	23	0.96 (1.56)		^**%**^**Hyper-insulinemia**			0.30
^**&**^**High SBP**			0.55	No	80	0.09 (1.39)	
No	80	0.08(1.25)		Yes	10	0.21 (4.86)	
Yes	10	0.24(14.6)		^**#**^**Insulin Resistance**			**0.06**
^**&**^**High DBP**			0.62	No	76	0.08(1.22)	
No	39	0.091(1.53)		Yes	14	0.27(18.29)	
Yes	51	0.02(1.35)					

^@^Mann–Whitney u Test or Kruskal–Wallis test

^&^ Normal Range according to metabolic syndrome definition in children

^**^Desired range reported in each person's body analyzer result sheet, was considered for classification of muscle mass (low-normal-high) and fat mass (low-normal-high)

^$$^ Classification for REE (slow-normal-fast) according to desirable ranges reported for each child in Indirect Calorimeter result sheet

^*^hs-CRP Reference Value according to sex and age. Normal value in serum (mg/liter): Boys: 5–13 year < 1.45 and 14–18 year < 2.13/Girls: 5–13 year < 1.90 and 14–18 year < 3.33

^#^ HOMA-IR cut-off values for insulin resistance were calculated to be 2.67 (sensitivity 88.2%, specificity 65.5%) in boys and 2.22 (sensitivity 100%, specificity 42.3%) in girls in the prepubertal period, and 5.22 (sensitivity 56%, specificity 93.3%) in boys and 3.82 (sensitivity 77.1%, specificity 71.4%) in girls in the pubertal period

**Table 7:** The comparison of miR-155 expression level among children with and without some metabolic status

Groups	*N*	Median (IQR)	^@^*P*-value	Groups	*N*	Median (IQR)	^@^*P*-value
**Obesity**			**0.01**	^**^Muscle Mass Range			0.13
No	45	0.37(0.61)		Low	41	0.4(0.54)	
Yes	45	0.17 (0.43)		Normal	24	0.30(0.58)	
**Metabolic syndrome**			0.10	High	25	0.18(0.39)	
No	76	0.33(0.52)		**Fat Mass			**0.03**
Yes	14	0.08(0.32)		Normal	55	0.36(0.60)	
^**&**^**High TG**			0.26	high	35	0.11(0.44)	
No	50	0.35(0.53)		^**$$**^**REE**			0.59
Yes	40	0.18(0.54)		^~^Slow	10	0.17(0.42)	
^**&**^**Low HDL**			0.51	^~^Normal	59	0.33(0.52)	
No	67	0.34(0.52)		^~^Fast	21	0.33(0.53)	
Yes	23	1.00(0.54)		*High hs-CRP			**0.02**
^**&**^**Large WC**			0.29	No	60	0.34(0.54)	
No	67	0.36(0.54)		Yes	29	0.10(0.33)	
Yes	23	0.17(0.39)		^**%**^**Hyper-insulinemia**			0.18
^**&**^**High SBP**			0.55	No	80	0.33(0.52)	
No	80	0.34(0.52)		Yes	10	0.09(0.16)	
Yes	10	0.19(0.30)		^**#**^**Insulin Resistance**			**0.06**
^**&**^**High DBP**			0.62	No	76	0.35(0.52)	
No	39	0.32(0.52)		Yes	14	0.06(0.16)	
Yes	51	0.12(0.60)					

^@^Mann–Whitney u Test or Kruskal–Wallis test

^&^ Normal Range according to metabolic syndrome definition in children

^**^Desired range reported in each person's body analyzer result sheet, was considered for classification of muscle mass (low-normal-high) and fat mass (low-normal-high)

^$$^ Classification for REE (slow-normal-fast) according to desirable ranges reported for each child in Indirect Calorimeter result sheet

^*^hs-CRP Reference Value according to sex and age. Normal value in serum (mg/liter): Boys: 5–13 year < 1.45 and 14–18 year < 2.13/Girls: 5–13 year < 1.90 and 14–18 year < 3.33

^#^ HOMA-IR cut-off values for insulin resistance were calculated to be 2.67 (sensitivity 88.2%, specificity 65.5%) in boys and 2.22 (sensitivity 100%, specificity 42.3%) in girls in the prepubertal period, and 5.22 (sensitivity 56%, specificity 93.3%) in boys and 3.82 (sensitivity 77.1%, specificity 71.4%) in girls in the pubertal period

The median (IQR) expression level of miR-122 was higher in children with metabolic syndrome, high-fat mass, high hs-CRP, larger WC, and insulin resistance significantly (for a review of *P*-values between two groups, cf Table 6). We found no significant difference in the miR-122 expression level of children with increased FBG, higher TG, higher SBP-DBP, lower HDL, hyperinsulinemia, and children with different REE with children who were in the normal range for these factors. Also, the expression level of miR-122 was not significantly different among children with different muscle mass. The median (IQR) expression level of miR-155 was significantly lower in children with high hs-CRP and high-fat mass compared to the normal group (for a review of P-values between two groups, cf Table 7). Similar analyses were done for miR-15a and miR-146 expression levels; we find no significant association between miR-15a and miR-146 with metabolic status, so the data were not reported.

## Discussion

Circulating miRNA concentrations have been suggested to be associated with a variety of metabolic diseases such as obesity and type 2 diabetes [20, 21, 45, 46]. Obesity-related miRNAs have been called potential biomarkers for the prevention and diagnosis of obesity and obesity-related metabolic disorders [46]. Obtained evidence also indicated that obesity-related miRNAs are promising new therapeutic tools for curing obesity and related diseases [46]. The current cross-sectional study was performed to compare the expression levels of miR-193a, miR-122, miR-155, miR-15a, and miR-146a, in peripheral blood mononuclear cells of obese children and adolescents with normal weight groups. The relation of expressed miRNAs with fasting insulin, HOMA-IR, fasting blood glucose, lipid profile and some inflammatory biomarkers were also assessed. Furthermore, the relation of differentially expressed miRNAs with some metabolic status (including metabolic syndrome, different body composition, hyper-insulinemia, insulin resistance, REE and high hs-CRP was evaluated.

Among the five studied miRNAs, obese children significantly had lower miR-155 than normal-weight children. After disaggregating the data by gender, the observed relationship was in place only for girls. MiR-155 had a negative association with fasting insulin, HOMA-IR, and hs-CRP. Mahdavi et al. showed that obese non-diabetic subjects had lower serum levels of miR-155 than normal-weight non-diabetic individuals [33]. Mazloom et al. reported that the expression of miR-155 in PBMC cells of diabetic patients was reduced compared to the control group [47]. They also reported a negative association between miR-155 and BMI, serum cholesterol, fasting insulin, HOMA-IR, and WC in the diabetic group [47]. According to previous studies, miR-155 plays a crucial role in the metabolism of cholesterol, and fatty acids in the liver through a direct effect on the regulatory factor X receptor alpha (LXRa). LXRa is involved in the regulation of many genes that contribute to fat metabolism [30, 31]. In animal models, reduction of miR-155 levels increases the risk of NAFLD in diabetic patients. In return, the miR-155 increase has a protective role in slowing NAFLD progression [31]. In line with these observations, in our study, the miR-155 was significantly lower in children with high fat-mass and insulin resistance compared to normal children.

In the present study, although the expression levels of miR-193a and miR-122 were higher in obese children, these observations were statistically significant only for Mir122 in girls. We found no previous study that compared the miR-193a expression level of obese children and adolescents with normal-weight controls, but similar studies had been done on adult participants. Some studies reported blood levels of miR-193a and miR-122 are elevated in participants with obesity, pre-diabetes, diabetes, insulin resistance, liver damage, and cardiovascular disorders significantly [48–54]. The possible reasons for this inconsistency could be due to different participant ages, the low sample size, and the metabolic phenotype of the studied children than the other studies. In this study, 55% of obese children did not have any obesity associated metabolic disorders like as metabolic syndrome and insulin resistance, While this percentage has been reported as 20-30% in other previous studies [7, 9]. Hess et al. (2020) assessed the serum levels of some miRNAs before and after a weight loss diet [55]. They showed that serum levels of miR-122 and miR-193a were reduced after an average weight loss of 5.7 Kg. They also demonstrated the levels of these miRNAs were positively correlated with metabolic syndrome, serum insulin, HOMA-IR, BMI, waist circumference, lean body mass, and visceral adipose tissue at the baseline. MiR-122 and miR-193a also had a positive correlation with TG level and fat mass, respectively [55]. In a recent study, serum levels of miR-29a and miR-122 were compared between obese with T2DM, obese without T2DM, and normal-weight children [56]. They also reported the positive relationship between the assessed miRNAs and waist circumference, BMI, TG, insulin, HOMA-IR, IL-6, hs-CRP, and TNF-a [56]. Wang et al. showed serum levels of miR-122 had a significant direct correlation with BMI, triglyceride, and HOMA-IR index, and a significant inverse correlation with HDL cholesterol levels [24]. Also, in this study, miR-122 was associated with an increased odds of insulin resistance in humans. Therefore, they suggested circulating miR-122 levels may act as a marker of obesity and insulin resistance [24]. Lischka et al. (2021) reported that miR-122 is positively correlated with HOMA-IR, TG, cholesterol and ALT as metabolic biomarkers and TNF-α, IL-1Ra, and pro-calcitonin as inflammatory biomarkers in pediatric patients. They also reported positive correlation of miR-193b with cholesterol, ALT, and MRI PDFF (magnetic resonance imaging-proton density fat fraction). On the other hand, miR-122 and miR-193b had no significant association with CRP and IL-6. Moreover, Lischka et al. revealed miR-122 expression level of patients with pre diabetes, impaired glucose tolerance or metabolic syndrome was significantly different from those obese children without these conditions [57].

In line with the mentioned studies, in the current study, miR-193a and miR-122 had a significant positive association with fasting insulin level, HOMA-IR, and TG. There was also a positive significant association between miR-122 and hs-CRP. The following comparative analysis conducted between these two miRNAs and some metabolic status showed expression levels of both were higher in children with metabolic syndrome, insulin resistance, and high-fat mass. Furthermore, expression levels of miR-193a and miR-122 were also higher in children with fast REE (than slow REE) and children with high hs-CRP, respectively.

Also, the present study could not find a significant difference in the expression of miR-15 between the two groups. However, previous studies have shown that miR-15 expression is reduced in type 2 diabetes, metabolic syndrome, and obesity, thereby miR-15 is known as a diagnostic biomarker for obese children at risk of type 2 diabetes mellitus [2]. Lischka et al., in 2021 reported miR-15a expression level was significantly lower in IGT pediatric patients [57]. In the current study, miR-15a had a significant association with serum insulin and HOMA-IR. this result was in line with previous observations regarding miR-15a importance as a predictor of diabetes in obese people regarding down-regulation of miR-15a in hyperglycemic conditions [58]. In the present study, none of the children had FBS higher than the normal range (neither obese children nor normal weight children), which may be the reason for observing no significant difference in the miR-15a level between the two study groups. In the present study, there was no significant difference between the two groups for the expression of miR-146a. Chartoumpekis et al. compared the expression levels of 530 miRNAs in adipose tissue of mice feeding a standard diet and mice feeding a high-fat diet. For the first time, they showed that miR-146a, miR-146b, and miR-379 were up-regulated during the development of obesity [59]. Ortego et al. reported that obese participants after Bariatric Surgery lost about 30 percent of their original weight and miR-146a was up-regulated by about 146% [60]. Sharma et al. designed a case-control study to investigate the relation of adipose tissue miRNAs with insulin sensitivity. They couldn’t find a significant difference in miR-146 expression level between the participants with and without insulin resistance, but the miR-146a and miR-146b had a significant correlation with body fat percentage, BMI, and insulin sensitivity index [29].

A few shortcomings of the study that can be improved in the future is to have a group of overweight children included to compare with obese and normal-weight children. Secondly, due to the cross-sectional design of the study cause and effect relationship could not be marked. Therefore, planning further studies to assess cause-effect correlations seems essential. For future studies, it is recommended that participants be categorized into two groups, metabolically healthy obese and metabolically unhealthy obese. Besides, considering groups of obese children with diabetes and metabolic syndrome will help grasp the affiliation of miRNAs with these diseases.

## Conclusions and future directions

In conclusion, among the five studied miRNAs (miR-193a, miR-122, miR-155a, miR-15a, and miR-146), only the expression level of miR-155 was significantly different in PBMC of obese children compared to the normal group (lower in obese children). Moreover, the expression level of miR-155 was negatively associated with fasting insulin and HOMA and also significantly lower in children with high hs-CRP and high-fat mass compared to the normal group. MiR-193a and miR-122 were positively correlated with insulin, HOMA, and TG. MiR-122 also was correlated with hs-CRP. After disaggregating the data by gender, the expression levels of miR-122 were higher in obese girls than normal-weight girls.

The expression levels of both miR-122 and miR-193a were higher in children with metabolic syndrome, insulin resistance, and high fat mass. Furthermore, the expression levels of miR-193a and miR-122 were also higher in children with fast REE (than slow REE) and high hs-CRP, respectively. Despite the limitations of our study, expression levels of miR-193a, miR-122, and miR-155a seem to be associated with some metabolic statuses, including obesity, metabolic syndrome, hyperinsulinemia, insulin resistance, and hyperlipidemia in obese children. Thereby, using the expression pattern of miRNAs in obese children and adolescents as a predictive marker of adulthood chronic disease appears to be very helpful. Further studies are suggested to evaluate the relationships and underlying mechanisms to achieve therapeutic interventions.

## Data Availability

The datasets used and/or analyzed during the current study are available from the corresponding author on reasonable request.

## Abbreviations

PBMC: Peripheral blood mononuclear cells
REE: Resting energy expenditure
hs-CRP: High-sensitivity C-reactive protein
WC: Waist circumference
HC: Hip circumference
BMI: Body mass index
WHR: Waist to hip ratio
TG: Triglyceride
HDL-C: High-density lipoprotein cholesterol
CVD: Cardiovascular disease
FFQ: Food frequency questionnaire
IL: Interleukin
LDL-C: Low-density lipoproteins cholesterol
MetS: Metabolic syndrome
IR: Insulin resistance
FBG: Fasting blood glucose
HOMA-IR: The homeostatic model assessment of insulin resistance
TNF-α: Tumor necrosis factor-α
SBP: Systolic blood pressure
DBP: Diastolic blood pressure
MAQ: Modifiable Activity Questionnaire
CDC: Charts of centers for disease control and prevention
GOI: Gene of interest
RQ: Relative quantities
NRQ: Normalized relative quantity
